# Ética, Inteligência Artificial e Cardiologia

**DOI:** 10.36660/abc.20200143

**Published:** 2020-09-18

**Authors:** Erito Marques de Souza, Fernando de Amorim Fernandes, Nikolas Cunha de Assis Pereira, Claudio Tinoco Mesquita, Ronaldo Altenburg Gismondi

**Affiliations:** 1 Universidade Federal Fluminense Niterói RJ Brasil Pós-graduação em Ciências Cardiovasculares da Universidade Federal Fluminense,Niterói, RJ - Brasil; 2 Universidade Federal Rural do Rio de Janeiro Departamento de Tecnologias e Linguagens Nova Iguaçu RJ Brasil Universidade Federal Rural do Rio de Janeiro - Departamento de Tecnologias e Linguagens,Nova Iguaçu, RJ – Brasil; 3 Setor de Medicina Nuclear do Serviço de Radiologia Hospital Universitário Antônio Pedro UFF Niterói RJ Brasil Setor de Medicina Nuclear do Serviço de Radiologia do Hospital Universitário Antônio Pedro (EBESERH-HUAP-UFF),Niterói, RJ - Brasil; 4 Faculdade de Medicina Universidade Federal Fluminense Niterói RJ Brasil Faculdade de Medicina da Universidade Federal Fluminense,Niterói, RJ - Brasil; 5 Hospital Niterói D´or Niterói RJ Brasil Hospital Niterói D´or,Niterói, RJ – Brasil

**Keywords:** Inteligência Artificial, Programas Informáticos, Tomada de decisões assistida por computador, Gestão de Informações

“De longe, o maior perigo da Inteligência Artificial é que as pessoas concluam muito cedo que a entendem. ”

Eliezer Yudkowsky

## Introdução

Em um futuro não muito distante, um programa de computador artificialmente inteligente provavelmente diagnosticará as doenças cardíacas com mais precisão que um cardiologista certificado. O conhecimento biomédico cresce significativamente, impossibilitando que os profissionais de saúde contemporâneos se mantenham atualizados sobre todos os conteúdos publicados em seu campo. Da mesma forma, informações sobre os pacientes estão cada vez mais numerosas e acessíveis, tornando impraticável o gerenciamento, filtragem e seleção em tempo real. Nesse contexto, a Inteligência Artificial (IA) tem um papel relevante na tomada de decisões em saúde. É resultado da combinação de modelos matemáticos sofisticados e da computação, para produzir algoritmos refinados capazes de emular (ou imitar) a inteligência humana.^1^ A IA permitiu aplicações interessantes em praticamente todos os campos da medicina e do conhecimento humano. Particularmente, na cardiologia, várias aplicações se mostraram exitosas. Han et al.,^2^ por exemplo, usaram o Machine Learning (ML), um subconjunto da IA, para analisar se essa ferramenta seria útil para identificar pacientes com risco de rápida progressão de placa coronariana. Foram utilizadas características epidemiológicas clínicas e informações quantitativas e qualitativas da angiografia tomográfica computadorizada das coronárias (todas obtidas a partir do estudo PARADIGM). No total, 1.083 pacientes foram incluídos no estudo, sendo testados 10 modelos diferentes. O LogitBoost apresentou melhor desempenho. A área sob a curva (AUC) *receiver operating characteristic* foi de 0,83 — melhor que o escore de risco de doença cardiovascular aterosclerótica de 10 anos (escore de risco DCA), que foi de 0,59. Em outro estudo, Than et al.,^3^ avaliaram se o *Gradient Boosting* (também um algoritmo de ML) seria benéfico na previsão da probabilidade de infarto agudo do miocárdio tipo 1. Foram considerados aspectos como sexo, idade, taxa de alteração da concentração cardíaca de troponina I e troponina cardíaca pareada I de uma amostra com 11.011 pacientes. A AUC foi de 0,96 e o modelo de ML teve melhor desempenho do que a rota tradicional da Sociedade Europeia de Cardiologia de 0/3 horas. Hedman et al.,^4^ desenvolveram um algoritmo de ML para descrever grupos de pacientes com insuficiência cardíaca com fração de ejeção preservada com base em seu fenótipo. Eles usaram dados clínicos e eletrocardiográficos. Foram identificados seis grupos diferentes, com diferentes níveis de proteínas inflamatórias e cardiovasculares e também com diferentes desfechos. Diante disso, na cardiologia, o processo de incorporação da IA na prática clínica é acelerado. O uso da IA na cardiologia está presente em nossa rotina diária, como o reconhecimento de fenótipos de doenças, diagnóstico, prognóstico e algoritmos de tratamento. A IA tem um enorme potencial disruptivo e alguns defendem a possibilidade do surgimento de uma nova espécie, o Homo incredibile^1^, que apoia suas decisões sobre dados e promove uma revolução no ecossistema digital. No entanto, essa mudança de paradigma trouxe inúmeros desafios, infelizmente. Questões éticas são uma grande preocupação em relação a essas novas tecnologias, e discutiremos algumas delas, bem como possíveis soluções e precauções.

### Aspectos Éticos

#### Discriminação e Privacidade de Dados

Esses algoritmos podem, por exemplo, ser usados para discriminar pessoas, dar vida a dispositivos que colocam outras vidas em risco ou até para produzir e divulgar notícias falsas — sem falar dos possíveis danos em caso de políticas inadequadas de segurança da informação.^5-7^ O sequestro de arquivos, ocorrido em 2017, com mais de 300 milhões de computadores atacados pelo ransomware WannaCry em 150 países e o vazamento de dados pela empresa Ashley Madison em 2015, são exemplos do potencial destrutivo de ações de hackers. Isso exemplifica alguns obstáculos a serem superados na inclusão de dispositivos médicos portáteis na prática clínica e no uso de sistemas autônomos para apoiar a tomada de decisões na área da saúde.

A obtenção do consentimento informado é uma preocupação da maioria dos bioeticistas. Os modelos atuais de IA dependem muito das informações contidas nos prontuários médicos. É possível garantir que as informações pessoais permaneçam confidenciais, mesmo com dados que circulam pela internet? O vazamento de informações médicas de pessoas famosas, como a ex-primeira-dama Marisa Silva e o atual Presidente, são apenas alguns exemplos de problemas relacionados à confidencialidade de dados.

#### Transparência e Segurança

Além disso, quando se trata de ciências médicas e da saúde, especificamente, outros riscos se destacam. Um deles é a falta de transparência na tomada de decisões ou a incapacidade de explicar o “raciocínio” por trás do resultado final, representado pelas chamadas caixas pretas. Foram feitas pesquisas em busca de soluções. No entanto, a realidade atual é que os responsáveis pela maioria das grandes coisas feitas com o *Deep Learning* não sabem explicar completamente o funcionamento de seus eficientes sistemas.^8-10^ Por outro lado, nem sempre é possível fornecer explicações detalhadas sobre a fisiopatologia de certas doenças ou sobre o mecanismo de ação de alguns medicamentos, mesmo que os ensaios clínicos tenham mostrado benefícios para o paciente. Isso aumenta o desafio de garantir a reprodutibilidade e a replicabilidade dos algoritmos de IA. Como apontado por Beam et al., um estudo é reproduzível se, com base no acesso a dados e na análise do código do algoritmo, um grupo independente puder obter os mesmos resultados observados no estudo original, enquanto a replicabilidade está associada ao fato de um grupo independente conseguir estudar o mesmo fenômeno e obter as mesmas conclusões após a realização de um conjunto de experimentos ou análises de um novo conjunto de dados.^11^ Outras questões relevantes são a segurança dos dados dos pacientes e a conscientização dos pacientes sobre o uso de seus dados. Uma parceria entre o Sistema Nacional de Saúde Britânico (NHS) e uma subsidiária de uma grande empresa privada de tecnologia em 2015, que incluiu a transferência não consentida de uma base de dados identificável de mais de 1,6 milhão de habitantes, é um dos casos mais famosos e controversos até o momento. Apesar das boas intenções de ambos os lados, ficou claro o quanto podemos estar expostos se não discutirmos, agora, até que ponto os dados pertencem a um indivíduo. Além disso, com esse tipo de contrato, grandes empresas de tecnologia tendem a aumentar ainda mais o oligopólio existente.

#### Valores e Preferências do Paciente, Julgamento Clínico e Empatia

O contato humano entre médicos e pacientes é um dos fundamentos da medicina desde Hipócrates. Existem dúvidas sobre se a IA é capaz de levar em consideração o contexto social da pessoa, fatores ambientais, preferências e valores morais no algoritmo de decisão do tratamento.

Outro aspecto importante é a representação de minorias étnicas, sociais e culturais nos prontuários médicos que servem de base para o algoritmo da IA. Se esses dados não forem muito representativos ou forem distorcidos, poderão ocorrer erros de interpretação.

## Medidas a Serem Implementadas

Conforme definido por Keskinbora,^12^ para confiar na IA, precisamos do seguinte:

- Transparência de dados, operação e algoritmos
- Credibilidade e auditabilidade, incluindo o relatório de vieses e erros
- Confiabilidade, com IA clinicamente validada
- Recuperabilidade, permitindo o controle manual da operação, se necessário

**Figura 1:**
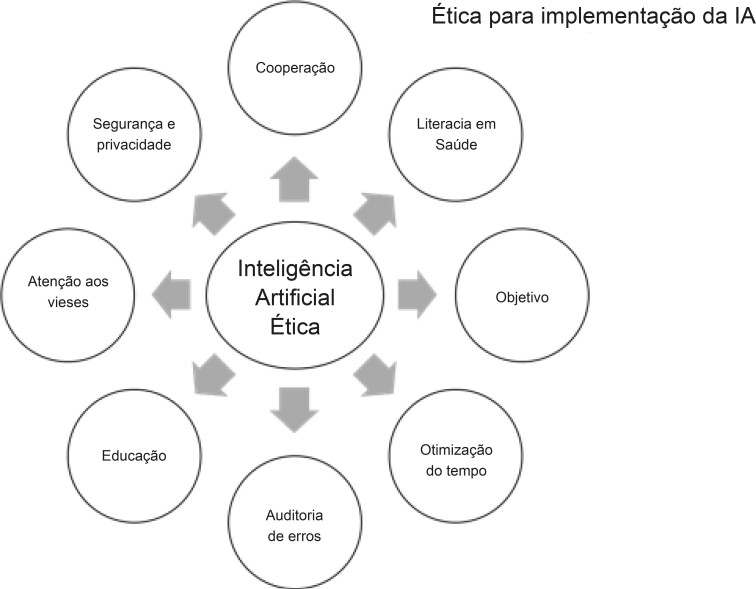
– Estratégia proposta para implementação da Inteligência Artificial na prática clínica considerando a ética. IA: inteligência artificial.

Esse cenário traz consigo a necessidade de discussão sobre o uso da IA na saúde, e seus limites, considerando os princípios fundamentais da bioética na saúde: justiça, não maleficência, beneficência, equidade, igualdade, aceitação social e respeito à autonomia do paciente.^13^ A questão que surge nesse contexto é: como incorporar a IA à prática biomédica, respeitando esses princípios para gerar valor? Embora não haja resposta definitiva para a pergunta, uma estratégia promissora (Figura 1) inclui:

- **Cooperação:** Os modelos de IA tendem a ter desempenho melhor quando temos dados saudáveis sobre o que queremos estudar. Assim, a colaboração interinstitucional exerce papel fundamental nesse processo, pois o compartilhamento desses dados favorece a obtenção de métricas de excelência.^1^
- **Literacia em Saúde:** refere-se ao nível de informações de saúde que cada indivíduo é capaz de obter, gerenciar e entender para aplicar no processo de tomada de decisões em saúde.^14^ Indivíduos com maior nível de literacia tendem a tomar melhores decisões em saúde. Assim, à medida que os modelos de IA são incorporados à prática clínica, é essencial que a literacia sobre eles também seja ampliada. Isso inclui uma relação médico-paciente ampliada, preocupada em incluir o paciente no centro da tomada de decisão multifatorial e multiprofissional. Da mesma forma, a literacia dos profissionais de saúde em IA deve ser incentivada.
- **Segurança e privacidade:** os dados criptografados são apenas o primeiro passo em medidas mais gerais para garantir a privacidade dos dados. O escândalo da Cambridge Analytica foi um grande aviso sobre os possíveis danos causados pelo mau uso dos BIG DATA. Nesse contexto, o estrito cumprimento do GDPR^15^ deve ser visto como um direito fundamental de qualquer ser humano, para o qual nenhum esforço deve ser feito para garantir. Essa é uma das questões mais centrais na referência ética para a implementação da IA e precisa estar bem estabelecida. Os prontuários médicos eletrônicos são os dados mais valiosos administrados por médicos e cardiologistas nos dias de hoje. Outra questão importante é a proteção das imagens fotográficas dos pacientes na medida em que se aplicam à tecnologia de reconhecimento facial, o que pode ameaçar o consentimento informado adequado e a segurança dos pacientes.^16^
- **Objetivo:** A IA deve ser usada como uma ferramenta cujo objetivo é promover a qualidade de vida, a saúde e o bem-estar dos seres humanos. Permitir que os interesses econômicos superem as reais necessidades humanas é um erro grave que pode ter consequências desastrosas. Keskinbora^12^ sugere, por exemplo, o desenvolvimento de uma IA livre, criada com um objetivo comum e cujo sentimento se baseie na operação de acordo com valores éticos.
- **Otimização do tempo:** o modus operandi da força de trabalho traz consigo tarefas que requerem inúmeras horas dos seres humanos em seu trabalho, realizando tarefas repetitivas. Muitas dessas tarefas podem ser substituídas por máquinas com desempenho melhor ou semelhantes aos humanos. Isso cria uma oportunidade na qual os seres humanos têm potencial para trabalhar menos, em empregos mais especializados. O tempo extra que eles têm deve ser investido em estudos adicionais, atividades de lazer, atividade física, dedicação à família etc. Isso certamente implica uma nova reformulação das políticas trabalhistas e educacionais.
- **Auditoria de erros e vigilância pública:** Os modelos de IA podem cometer erros e suas decisões nem sempre são compreensíveis para os seres humanos. Portanto, é essencial que os algoritmos sejam auditados periodicamente e que suas métricas de desempenho sejam informadas aos pacientes antes de tomar decisões sobre sua saúde. Há um desafio importante aqui, que está relacionado ao desenvolvimento de um aparato jurídico específico sobre o assunto.
- **Educação:** ao longo de sua vida, diferentes conjuntos de conhecimentos humanos se tornam inúteis. Em um mundo volátil, instável, complexo e ambíguo, as tecnologias disruptivas podem tornar obsoletas as habilidades e os conhecimentos anteriores ao longo do tempo: é a meia-vida do conhecimento biomédico. A solução é o processo de estudo contínuo, onde os seres humanos estudarão para sempre! Outro ponto é o quê estudar; certamente, um modelo centrado na memória deve ser substituído por um modelo voltado para a solução de problemas reais da sociedade. Para tanto, é obrigatório ampliar os estudos de matemática, computação e ciências básicas nos cursos de graduação e pós-graduação em saúde com esse objetivo.^1^
- **Atenção aos vieses:** Os modelos de Machine Learning (um subconjunto da IA) produzem suas respostas de acordo com os dados que são usados como entradas para os algoritmos. Assim, é possível gerar comportamento discriminatório em relação a determinados grupos. Por exemplo, se não houver dados suficientes para serem usados no treinamento de algoritmos. Um exemplo desse viés foi o *chatbot* do programa Tay da Microsoft, que aprendeu linguagem racista e sexista e precisou ser removido no dia do seu lançamento.^17,18^

## Exemplo de Caso

Veja o seguinte exemplo hipotético. Um homem de 70 anos sofre de insuficiência cardíaca: dispneia ao esforço, ortopneia, crepitações nas bases pulmonares e edema dos membros inferiores. Preocupado com sua situação, acessou rapidamente um site de diagnósticos médicos online, que mostrou 99% de probabilidade de insuficiência cardíaca. Para economizar tempo, ele realizou um ecocardiograma sozinho. Um algoritmo de diagnóstico de IA mostrou 97% de chances de cardiomiopatia dilatada idiopática, com prognóstico de 12 meses de sobrevida de apenas 13% e contraindicação ao transplante. E isso veio escrito no prontuário automático gerado por um computador com IA.

Chateado com a situação, ele vendeu todos os seus pertences e reservou uma viagem pelos cinco continentes para o mês seguinte, mas a companhia aérea exigiu um atestado médico, alegando que havia 80% de chance de complicações a bordo, além de cobrar uma taxa extra de 30% sobre o valor final. A empresa de seguros de viagem não quis oferecer uma apólice de seguro ao paciente com base em seu perfil de risco e a companhia de seguros de saúde foi à justiça para rescindir o contrato, pois seu relógio de pulso indicava um período de arritmias ventriculares que o paciente negara ao assinar o contrato.

No entanto, na consulta médica, o médico constatou que o paciente havia nascido em uma área de risco para a doença de Chagas, um dado que havia sido omitido. Como resultado, foi realizada a sorologia Elisa, que permitiu o início do tratamento e atrasou a progressão da doença.

Ao ler esse trecho, que possíveis usos indevidos da IA foram identificados?

Privacidade de dados, respeito à autonomia, erros de entrada de dados, viés do algoritmo de diagnóstico. Você acha isso muito difícil de acontecer? Algumas pessoas ainda pensam que a conexão de vídeo e voz é uma cena dos Jetsons!

## Conclusões

A IA certamente traz uma possível revolução na área da saúde. No entanto, seu uso inadequado pode ser uma fonte prejudicial para os pacientes. Os preceitos éticos devem, portanto, ser o pilar norteador de qualquer implementação dessa tecnologia. Uma questão importante que deve sempre ser fundamental na prática clínica é a empatia; a capacidade de entender ou sentir o que outra pessoa está passando a partir do ponto de vista dela. Os cardiologistas precisam usar suas habilidades clínicas, sua sabedoria, empatia e princípios éticos para usar ferramentas de assistência baseadas em inteligência artificial no melhor interesse de seus pacientes. Nesse contexto, o reconhecimento e a identificação de vulnerabilidades e desafios associados ao tema devem fazer parte do cotidiano das instituições de saúde.
