# Supplementary material for: Ética, Inteligência Artificial e Cardiologia
Source: Arq Bras Cardiol. 2020 Sep 18;115(3):579–83. [Article in Portuguese] doi: 10.36660/abc.20200143 (PMC9363099; doi:10.36660/abc.20200143)
Supplement: Translation

# Translation: Ethics, Artificial Intelligence and Cardiology

**Authors:** Erito Marques de Souza, Fernando de Amorim Fernandes, Nikolas Cunha de Assis Pereira, Claudio Tinoco Mesquita, Ronaldo Altenburg Gismondi

**Affiliations:** 1 Brazil Pós-graduação em Ciências Cardiovasculares da Universidade Federal Fluminense, Niterói, RJ - Brazil; 2 Brazil Universidade Federal Rural do Rio de Janeiro - Departamento de Tecnologias e Linguagens, Nova Iguaçu, RJ – Brazil; 3 Brazil Setor de Medicina Nuclear do Serviço de Radiologia do Hospital Universitário Antônio Pedro (EBESERH-HUAP-UFF), Niterói, RJ - Brazil; 4 Brazil Faculdade de Medicina da Universidade Federal Fluminense, Niterói, RJ - Brazil; 5 Brazil Hospital Niterói D´or, Niterói, RJ – Brazil

**Keywords:** Artificial Intelligence, Software, Decision Making,Compter-Assisted, Information Management

“By far, the greatest danger of Artificial Intelligence is that people conclude too early that they understand it.”

Eliezer Yudkowsky

## Introduction

In the not too distant future, an artificially intelligent computer program will probably diagnose heart conditions more accurately than a board-certified cardiologist. Biomedical knowledge grows significantly, making it impossible for contemporary health professionals to be updated on all content published in their field. Similarly, the amount of information about the patient is increasingly larger and more accessible, making real-time management, filtering and selection impractical for an individual. In this context, Artificial Intelligence (AI) has a relevant role in health decision-making. It is the result of the combination of sophisticated mathematical models and computation to produce refined algorithms capable of emulating (or imitating) human intelligence.^1^ It has allowed for interesting applications in virtually all fields of medicine and human knowledge. In particular, in cardiology, several applications have been shown to be successful. Han et al.,^2^ for instance, used machine learning (ML), a subset of AI, to analyze if this tool would be helpful to identify patients at risk of future rapid coronary plaque progression. Clinical epidemiological features and quantitative and qualitative information from coronary computed tomographic angiography were used (all of which were obtained from the PARADIGM study). They included 1,083 patients in the study and tested 10 different models. LogitBoost performed better. The area under the receiver operating characteristic curve (AUC) was 0.83 — better than the 10-year atherosclerotic cardiovascular disease risk score (ASCVD risk score), which was 0.59. In another study, Than et al.^3^ evaluated whether Gradient Boosting (also a ML algorithm) would be beneficial in predicting the likelihood of type 1 acute myocardial infarction. Features such as sex, age, rate of change of cardiac troponin I concentration and paired cardiac troponin I of a sample with 11,011 patients were considered. AUC was 0.96 and the ML model had better performance than the traditional European Society of Cardiology 0/3-hour pathway. Hedman et al.^4^ developed a ML algorithm to describe heart failure with preserved ejection fraction groups of patients based on their phenotype. They used clinical and electrocardiogram data. Six different groups were identified, with different levels of inflammatory and cardiovascular proteins and also with different outcomes. In light of that, in cardiology, the process of incorporating AI into the clinical practice is accelerated. The use of AI in cardiology is present in our daily lives, such as recognition of disease phenotypes, diagnosis, prognosis, and in treatment algorithms. AI has a huge disruptive potential and some advocate the possibility of the emergence of a new species, Homo incredibile,^1^ which supports its decisions on data and promotes a revolution in the digital ecosystem. However, this paradigm shift has unfortunately brought with it a myriad of challenges. Ethical issues are a great concern regarding these new technologies, and we will discuss some of them, as well as possible solutions and precautions.

### Ethical Concerns

#### Discrimination and Data Privacy

These algorithms can, for example, be used to discriminate against people, to give life to devices that put other lives at risk or even to produce and disseminate fake news — not to mention the potential damage in case of inadequate information security policies.^5-7^The hijacking of files that took place in 2017, with more than 300 million computers affected by the WannaCry ransomware in 150 countries and the data leak by the company Ashley Madison in 2015, are examples of the destructive potential of hacker actions. This exemplifies some obstacles to be overcome on the inclusion of wearable medical devices in clinical practice and on the use of autonomous systems to support decision-making in healthcare.

Obtaining informed consent is a concern of most bioethicists. Current models of AI are very dependent on information from medical records. Is it possible to ensure that personal information remains confidential even with data traveling over the internet? Leakage of medical information of famous people such as former First Lady Marisa Silva and the current President are just a few examples of problems with data confidentiality.

#### Transparency and Safety

In addition, when it comes to the medical and health sciences, specifically, other risks stand out. One of them is the lack of transparency in decision-making or the inability to explain the “reasoning” to obtain the final result, represented by the so-called black boxes. Research has been carried out in search of solutions. However, the current reality is that those responsible for most of the great things done with Deep Learning do not know how to fully explain the functioning of their successful systems.^8-10^ On the other hand, it is not always possible to provide detailed explanations about the pathophysiology of certain diseases or about the mechanism of action of some drugs, even though clinical trials have shown benefits for the patient. This adds to the challenge of ensuring the reproducibility and replicability of AI algorithms. As pointed out by Beam et al., a study is reproducible if, based on data access and analysis of the algorithm code, an independent group can obtain the same results observed by the original study while replicability is associated with the fact that an independent group can study the same phenomenon and obtain the same conclusions after carrying out a set of experiments or analyses from a new set of data.^11^ Other relevant questions are how safe the patients’ data are and how aware the patients are about the use of their data. A partnership between the British National Health System (NHS) and a subsidiary of a big private tech company in 2015, which included the unconsented transfer of an identifiable database of more than 1.6 million inhabitants, was one of the most famous and controversial cases to date. Despite the good intentions of both sides, it was clear how much we can be exposed if we do not discuss, right now, the extent to which the data is owned by an individual. Moreover, with this type of agreement, large technology companies tend to further increase the existing oligopoly.

#### Patient Values and Preferences, Clinical Judgement and Empathy

Human contact between doctors and patients is one of the foundations of medicine since Hippocrates. Doubts exist as to whether an AI is able to take into account the person’s social context, environmental factors, preferences and moral values in the treatment decision algorithm.

Another important aspect is the representation of ethnic, social and cultural minorities in the medical records that serve as the basis for the AI algorithm. If these data are not very representative or skewed, errors of interpretation may occur.

#### Measures to be Implemented

As defined by Keskinbora,^12^ to trust AI we need the following:

- Transparency of data, operation and algorithms
- Credibility and auditability, including the report of bias and errors
- Reliability, with clinically validated AI
- Recoverability, allowing manual control of the operation if needed

This scenario brings with it the need for a discussion on the use of AI in health and its limits considering the fundamental principles of bioethics in health: justice, non-maleficence, beneficence, fairness, equality, social acceptance and respect for patient autonomy.^13^ The question that emerges in this context is: how to incorporate AI into biomedical practice while respecting these principles in order to generate value? Although there is no definitive answer to the question, a promising strategy (figure 1) includes:

**Figure 1:** – Proposed strategy for Artificial Intelligence implementation in clinical practice considering ethics. AI: artificial intelligence.

- **Cooperation**: AI models tend to perform better when we have healthy data about what we want to study. Thus, interinstitutional collaboration has a fundamental role in this process, as the sharing of this data favors the achievement of metrics of excellence.^1^
- **Health Literacy**: this relates to the level of health information that each individual is able to obtain, manage and understand to apply in the decision-making process of health.^14^ Individuals with greater literacy tend to make better health decisions. Thus, as AI models are incorporated into clinical practice, it is essential that literacy about them also be expanded. This includes an expanded doctor-patient relationship, which is concerned with including the patient at the center of multifactorial and multi-professional decision-making. Similarly, health professional literacy in AI should be encouraged.
- **Security and privacy**: encrypted data is just the first step in more general measures to ensure data privacy. The Cambridge Analytica scandal was a major warning about the potential harms due to the misuse of BIG DATA. In this context, strict compliance with GDPR^15^ should be seen as a fundamental right of any human being for which no efforts should be made to ensure. This is one of the most central issues in the ethical reference for AI implementation and needs to be well secured. Medical electronic records are the most valuable data clinicians and cardiologists are managing today. Another important issue is the protection of patient photographic images as they apply to facial recognition technology, which could threaten proper informed consent and safety of patients.^16^
- **Purpose**: AI should be used as a tool whose objective is to promote quality of life, health and well-being of human beings. Letting economic interests outweigh real human needs is a serious mistake that can have disastrous consequences. Keskinbora^12^ suggests, for example, the development of a free AI created with a common objective and whose feeling is based on the operation according to ethical values.
- **Time optimization**: the modus operandi of the workforce brings with it jobs that require a large number of hours from human beings in their work, performing repetitive tasks. Many of these tasks can be replaced by machines that perform better or like humans. This creates a window of opportunity in which human beings have the potential to work less, in more specialized jobs. The extra time they have should be invested in further studies, leisure activities, physical activity, family care, etc. This certainly implies a new reformulation of labor and educational policies.
- **Audit of errors and public surveillance**: AI models can make mistakes and their decisions are not always understandable to humans. Therefore, it is essential that the algorithms be audited periodically and that their performance metrics be informed to patients before making any decisions regarding their health. There is an important challenge here, related to the development of a specific legal apparatus on the subject.
- **Education**: throughout its life, different sets of human knowledge become useless. In a volatile, unstable, complex and ambiguous world, disruptive technologies can render obsolete previous skills and knowledge over time: it is the half-life of biomedical knowledge. The solution is the process of continuous study, where human beings study forever! Another point refers to what to study; certainly a model centered on memory should be replaced by a model focused on solving real problems in society. To this end, expanding the study of mathematics, computing and basic sciences in graduate and postgraduate courses in health with this objective is mandatory.^1^
- **Attention to biases**: Machine Learning models (a subset of AI) produce their responses according to the data that are used as inputs for the algorithms. Thus, it is possible to generate discriminatory behavior in relation to certain groups. For instance, if there is not enough data to be used in algorithm training. An example of this prejudiced bias was Microsoft Tay’s chatbot, which learned racist and sexist language and needed to be removed on the day of its launch.^17,18^

## Case Example

Consider the following hypothetical example. A 70-year-old man has a heart failure condition: dyspnea on exertion, orthopnea, crackles in the lung bases and edema of the lower limbs. Concerned about his situation, he made a quick visit to an online medical diagnosis website, which showed 99% probability of heart failure. In order to save time, he performed an echocardiogram on his own. An AI diagnostic algorithm showed 97% chances of having idiopathic dilated cardiomyopathy, with a prognosis of 12-month survival of only 13% and contraindication to transplantation. And that was written in the automatic report generated by a computer with AI.

Upset about the situation, he sold all of his belongings and booked a trip across five continents for the following month, but the airline demanded a medical certificate, claiming that there was an 80% chance of on-board complications, in addition to charging an extra fee of 30% on the final amount. The travel insurance firm did not want to offer an insurance policy to the patient based on his risk profile and the health insurance company went to court to break the contract, as his wristwatch had indicated a period of ventricular arrhythmias that the patient denied when he signed the contract.

However, at the medical appointment, the doctor found that the patient was born in a risk area for Chagas’ disease, which omitted. As a result, Elisa serology was conducted, which allowed the initiation of treatment and delayed the progression of the disease.

By reading this excerpt, what possible misuses of AI were identified?

Data privacy, respect for autonomy, data input errors, diagnostic algorithm bias. Do you find this very difficult to happen? Some people still think that the video and voice connection is a scene from the Jetsons!

## Conclusions

AI certainly brings a potential revolution in healthcare. However, its improper use can be a harmful source for patients. Ethical precepts must, therefore, be the guiding pillar of any implementation of this technology. An important issue that must be always key in clinical practice is empathy; the capacity to understand or feel what another person is experiencing from their point of view. Cardiologists need to use their clinical skills, wisdom, empathy and ethical principles to use artificial intelligence-based assistance tools in the best interest of their patients. In this context, the recognition and identification of vulnerabilities and challenges associated with the theme must be part of the routine of health institutions.
